# An Optically Pumped Magnetometer with Omnidirectional Magnetic Field Sensitivity

**DOI:** 10.3390/s23156866

**Published:** 2023-08-02

**Authors:** Volkmar Schultze, Theo Scholtes, Gregor Oelsner, Florian Wittkaemper, Torsten Wieduwilt, Ronny Stolz

**Affiliations:** Leibniz Institute of Photonic Technology Jena, Albert-Einstein-Straße 9, D-07745 Jena, Germany; theo.scholtes@leibniz-ipht.de (T.S.);

**Keywords:** magnetometer, optically pumped magnetometer, dead zone, heading error, intensity modulation, amplitude modulation, light shift, nonlinear Zeeman effect

## Abstract

In mobile applications such as geomagnetic surveying, two major effects hamper the use of optically pumped magnetometers: dead zones, sensor orientations where the sensors signal amplitude drops; and heading errors, a dependence of the measured magnetic field value on the sensor orientation. We present a concept for an omnidirectional magnetometer to overcome both of these effects. The sensor uses two cesium vapor cells, interrogated by circularly-polarized amplitude-modulated laser light split into two beams propagating perpendicular to each other. This configuration is experimentally investigated using a setup wherein the laser beam and magnetic field direction can be freely adjusted relative to each other within a magnetically shielded environment. We demonstrate that a dead-zone-free magnetometer can be realized with nearly isotropic magnetic-field sensitivity. While in the current configuration we observe heading errors emerging from light shifts and shifts due to the nonlinear Zeeman effect, we introduce a straightforward approach to suppress these systematic effects in an advanced sensor realization.

## 1. Introduction

Optically pumped magnetometers (OPMs) exploit the Zeeman effect in vapors of alkali atoms [1]. These sensors rely on creating atomic polarization by optical pumping and optical readout of the coherent Larmor precession of atoms within the magnetic field of interest. While such OPMs inherently infer the absolute value of the magnetic field vector, thus being termed “scalar”, typical implementations suffer from the dependence of sensor orientation on the ambient magnetic field direction. On one hand, the dependence of the signal amplitude on the sensor orientation can lead to a drop in sensor sensitivity in certain configurations (“dead zones”) [2]. On the other hand, there are effects that lead to systematic shifts in the sensor reading dependent on the sensor orientation. These effects, termed “heading errors”, can emerge from (vector) light shifts (also known as AC Stark shifts) or from shifts due to the nonlinear Zeeman effect. The latter is especially prominent in the geomagnetic field intensity range [3,4]. While such systematic effects may be calibrated for or even ignored in stationary applications of OPM systems, they become especially important for measurements within the Earth’s magnetic field, such as in magnetic field exploration [5]. In such applications, systematic effects typically outrange the sensor’s intrinsic sensitivity by several orders of magnitude, heavily limiting the overall sensor performance.

In the past, research has been devoted to understanding and reducing the heading errors and dead zones of OPMs. Some approaches exploit the fact that the shift caused by the nonlinear Zeeman effect changes sign with the helicity of the circularly polarized pumping light. This allows the cancellation of the heading error using two alkali vapor cells (or two separated compartments), which are optically pumped (and read out) by circularly polarized light propagating in opposite directions [6]. Alternatively, the heading error can also be canceled by light propagating collinearly with opposite circular helicity σ^+^ and σ^−^ [7]. Recently, a method termed “spin locking” has been developed, which uses amplitude-modulated light and an additional rf field to eliminate the asymmetric line splitting by the nonlinear Zeeman effect [8]. However, this approach was only tested within a small range of tilting angles of about 10°. Besides this, pumping with light changing back and forth from σ^+^ via linear to σ^−^ polarization at Larmor frequency was shown to suppress dead zones efficiently [9].

A straightforward approach to eliminate the dead zones involves using combinations of multiple OPM cells with different orientations concerning the magnetic field to be measured. For example, when using three orthogonally oriented M_z_ OPMs, under any orientation in the Earth’s magnetic field, at least one cell gives a magnetometer signal [10]. It was also shown that just two OPM cells are sufficient when using collinearly propagating modulated laser beams of two perpendicular linear polarizations [11]. Also, realizations with only one cell have been tested, requiring a mechanical rotation of the light polarization orientation [12,13] to track changing magnetic field orientations.

Here, we present the concept of an omnidirectional magnetometer based on two sensor compartments using amplitude-modulated light. In the next section, we introduce our realization and discuss its basic functionality and advantages. In Section 3, the experimental setup is described, after which we discuss measurement results and draw conclusions for further work.

## 2. Concept of the Omnidirectional OPM

**Figure 1:**
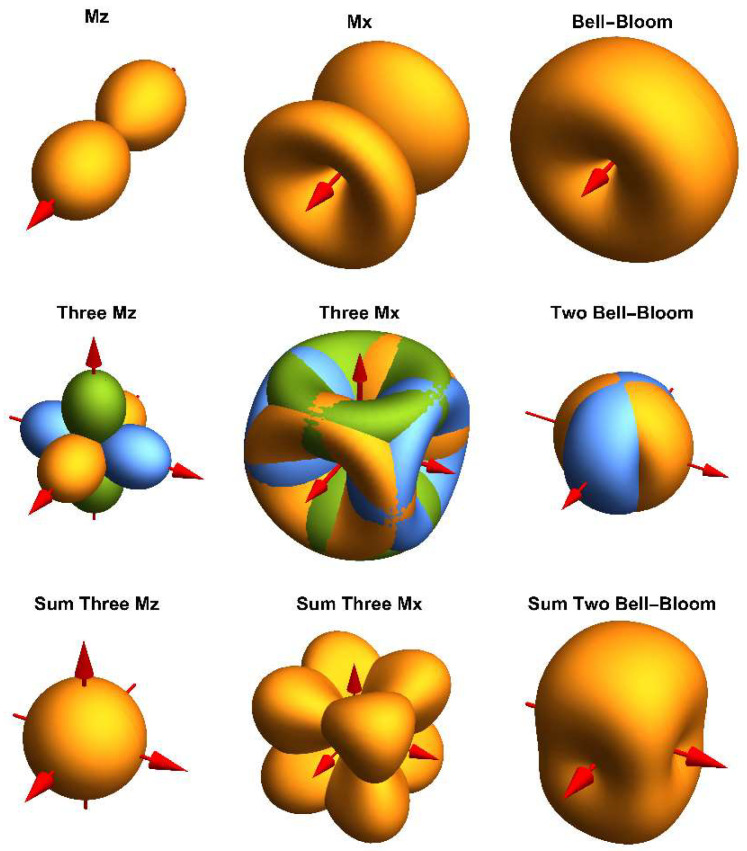
Dependence of the normalized signal amplitude of different OPM operational modes on the relative magnetic field orientation for single (**upper** row) and multiple (**middle** and **lower** row) cell arrangements. In the middle row, the signals of the individual cells are shown in different colors. The lower row shows their sum signal.

In the first analysis, we assessed the most promising operational modes for realizing an omnidirectional OPM. In all these modes, the atoms of an alkali vapor are pumped with circularly polarized light. Thereby, the electron spins are oriented along the propagation direction of the pumping laser beam ***k***. Due to interaction with the magnetic field ***B***_0_ the spins precess at Larmor frequency *f*_L_ = γ × ***B***_0_ (with γ being the gyromagnetic ratio of the probed hyperfine level of the alkali atom). The precession of the spins has to be synchronized in phase to be detectable. In M_x_ and M_z_ magnetometers, this is done with an additional magnetic field ***B***_1_ oscillating at Larmor frequency. While in the M_x_ scheme, the pumping light modulation amplitude or phase at the ***B***_1_ frequency is analyzed, in the M_z_ magnetometer, the change in dc light absorption is measured [14]. In the so-called “Bell-Bloom” (BB) magnetometers [15], phase synchronization is achieved by the modulation of the pumping light. These different configurations yield different spatial distributions of the sensitivity concerning the magnetic field vector ***B***_0_. Figure 1 shows calculated OPM signal amplitudes in dependence on the propagation direction of the pump light for these three modes.

In the M_x_ magnetometer, the signal depends solely on the so-called incident angle ξ between ***k*** and ***B***_0_, when ***B***_1_ is oriented parallel to ***k***. Then the (normalized) signal amplitude follows the spatial distribution [16]:

(1) $$M_{x}(\xi )=4\cdot \left|\cos(\xi )\right|\cdot \sin^{2}\left(\xi \right)\cdot \frac{\sqrt{2}}{1+2\cdot \sin^{2}\left(\xi \right)}$$

Even when using three mutually orthogonally arranged M_x_ magnetometers, there are certain sensor orientations (with respect to ***B***_0_) where the signal amplitude vanishes. This is due to the small funnel-shaped spatial amplitude distribution shown in Figure 1.

The spatial amplitude distribution of the M_z_ magnetometer [2]:

(2) $$M_{z}(\xi )={cos}^{2}(\xi )$$

has a dumbbell form and results from the projection of the magnetic field vector onto the light propagation direction ***k***. For three orthogonally arranged M_z_ magnetometers, the amplitudes for the three coordinate axes are explicitly given in Figure 1.

The red arrows denote the light propagation directions ***k*** of the laser beams.

(3) $${M_{z}}^{\left(x\right)}(\varphi ,\theta )={cos}^{2}(\varphi )\cdot {sin}^{2}(\theta )\;{M_{z}}^{\left(y\right)}(\varphi ,\theta )={sin}^{2}(\varphi )\cdot {sin}^{2}(\theta )\;{M_{z}}^{\left(z\right)}(\varphi ,\theta )={cos}^{2}(\theta )$$

Equation (3) follows from Equation (2) when a rotation around the respective coordinate axis is regarded. For M_z_^(x)^ and M_z_^(y)^ the relation between the angles φ and θ (the common spherical coordinates) and the incident angle ξ given by Equation (A4) can also be used. The sum of these signals describes a sphere. Therefore, as shown in Figure 1, such an arrangement would deliver a perfect omnidirectionally operating OPM. However, the M_z_ magnetometer has an inferior magnetic-field resolution compared to M_x_ [1]. Methods for strongly improved performance of M_z_ magnetometers have been suggested and tested [17,18]. However, these approaches require two cell compartments for each of the axes, pumped with two beams of circularly polarized light having opposite helicity. Hence, the effort of implementing such an omnidirectional OPM would be significant. This is because six laser beams with very high demands for light power and polarization balancing are required.

In contrast, the operation of an OPM with intensity-modulated (IM) pumping light (a special kind of the BB magnetometer [15]) features an even more beneficial dependence of signal amplitude on orientation angle [19]:

(4) $$IM^{\left(x\right)}(\varphi ,\theta )=1-\cos^{4}\left(\varphi \right)\cdot \sin^{4}\left(\theta \right)\;IM^{\left(y\right)}(\varphi ,\theta )=1-\sin^{4}\left(\varphi \right)\cdot \sin^{4}\left(\theta \right)$$

They have the great advantage of being sensitive in all directions except for the propagation direction ***k*** of the pumping laser light, thus covering two spatial dimensions with a single magnetometer (Figure 1). Thus, two already perpendicularly oriented BB magnetometers can cover the full solid angle. As no ***B***_1_ field is necessary, the BB sensor is “magnetically silent”, preventing potential inter-channel crosstalk [20]. Typically, in such OPMs, the detection of the spin precession is implemented using an additional probe beam, typically oriented perpendicularly or at least inclined concerning the pumping beam [19]. However, we have shown that, when using the pumping laser simultaneously for probing, a magnetic-field resolution comparable to the M_x_ magnetometer can be achieved [21]. This one-beam configuration is not only the simplest one but has another decisive advantage for an omnidirectional measurement. As was shown in [22], a deviation of the probe beam direction from the pumping beam produces a phase shift depending on the magnetic field direction, thus leading to a systematic error in the measured Larmor frequency.

We conclude that a configuration with two perpendicularly arranged intensity-modulated magnetometers using only one beam for both pumping and probing represents the most promising approach to omnidirectional operation considering dead zones, heading error, and sensor complexity, as well as expected magnetic field sensitivity.

## 3. Measurement Method

### 3.1. Experimental Setup

**Figure 2:**
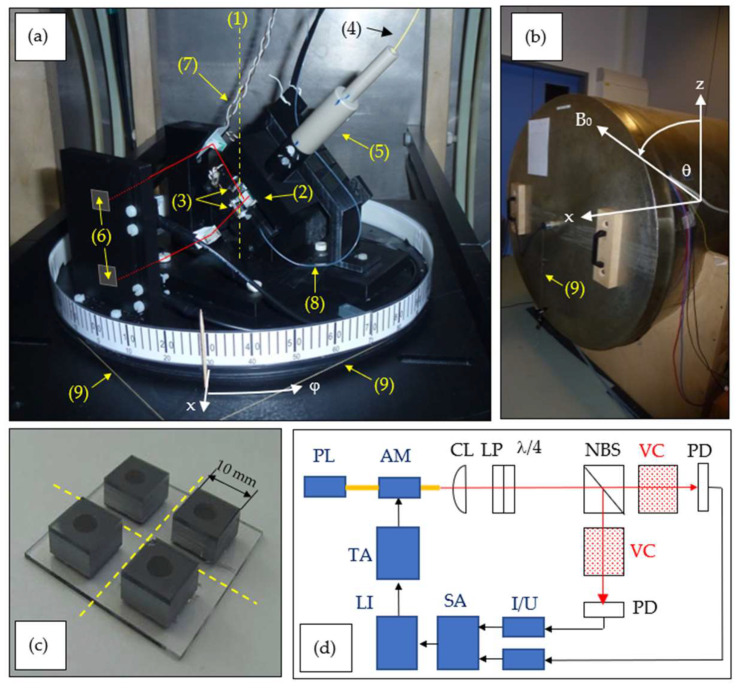
Measurement setup.

For the experimental investigation of the omnidirectional configuration, we adapted a setup that has already been used for heading error investigations of OPMs [3]. It is based on a turntable on which the vapor cells and required optical components are mounted, as shown in Figure 2a.

The turntable is mounted inside a three-layer magnetic shielding barrel (Figure 2b), including a three-axis Helmholtz coil system for applying magnetic fields in arbitrary directions [23]. We ensured that the vapor cells were both centered on the rotational axis of the turntable by placing them at an inclination of ±45° with respect to the turntable’s *xy* plane. This way, during the rotation of the turntable, the influence of remaining magnetic field gradients in the *xy* plane is eliminated. The two vapor cells are at a distance of 15 mm in the *z*-direction (vertical rotational axis).

**Figure 3:**
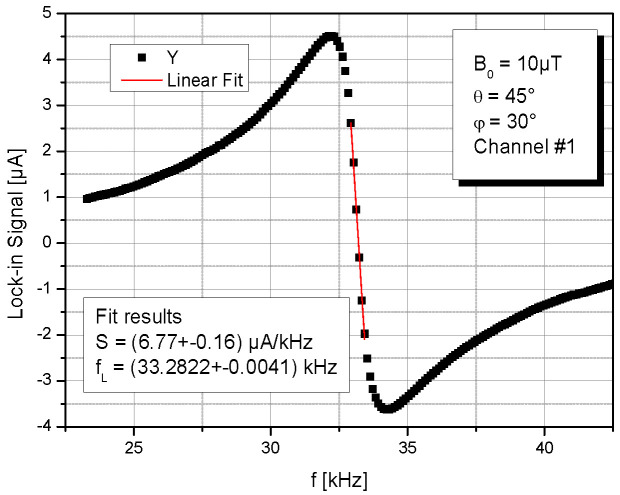
Example for a dispersive lock-in signal *Y* with linear fit (red curve) of the inner range.

The cesium cell cavities are realized by ultrasonic milling of thru-holes with a 4 mm diameter into 4 mm thick silicon wafers enclosed by anodically bonded glass plates [24]. The cells feature nitrogen buffer gas pressures of 38 mbar (cell #1) and 28 mbar (cell #2), respectively. These slightly different values result from the photolytic decomposition of cesium azide in the individual cells. Thin-film heaters attached on the sides of the cells’ silicon parts are fed with an AC current of 10 kHz, a frequency well distinct from the Larmor frequency and well above the measurement bandwidth of about 500 Hz. The bandwidth value was inferred from the OPM’s frequency-dependent response to an AC magnetic field test signal. In the used free-running measurement mode (i.e., without the feedback of the light-modulation frequency to the actual Larmor frequency), it is determined as half of the resonance width (Figure 3).

The laser radiation for optical pumping and sensor readout from a distributed Bragg reflector (DBR) laser (λ = 894.6 nm) is delivered using individually assembled single-mode bow-tie fibers because of their better robustness against mechanical movement and temperature changes as compared to more commonly used panda fibers. The light intensity is square-wave modulated at full modulation depth with a 50% duty cycle using a fiber-coupled integrated Mach-Zehnder interferometer. The high-intensity value is varied to attain the best magnetic field resolution.

In the first set of experiments, we optimized the experimental parameters for the lowest shot-noise limited field resolution *B*_sn_. A magnetic field of 10 μT is used, yielding a Larmor frequency near 35 kHz. It is applied along the *x*-axis (θ = 90°). With an initial turntable orientation of φ = 0°, the pumping light direction ***k*** is perpendicular to ***B***_0_ for both OPM cells. For this case, the best shot-noise limited resolution of *B*_sn_ ≈ 100 fT/√Hz is achieved at cell temperatures of 60 °C and a pumping power in front of the cells of 170 µW. The laser light frequency was set about 1 GHz higher than at the buffer-gas broadened and shifted *F* = 4 → *F*′ = 3 hyperfine transition of the Cs D1 line. This configuration was found to optimize the shot-noise limited sensitivity *B*_sn_. These parameters were fixed throughout all further measurements. Only the relative orientation of pumping light direction ***k*** and magnetic field ***B***_0_ was varied.

(a) Turntable inside magnetic shielding barrel. (1) rotation axis (*z*-axis); (2) non-polarizing beam splitter; (3) the two cesium vapor cells, where the lower and upper cell are further labeled as #1 and #2, respectively; (4) optical fiber delivering laser light; (5) light collimation and polarization conditioning with lens, linear polarizer and λ/4 wave plate; (6) two photodiodes (hidden by their plastic mount); (7) wires connecting to the thin-film heaters; (8) fiber-based temperature sensor; (9) pull strings for manual table turning. The laser beam paths are sketched in red.
(b) Closed magnetic shielding barrel with turntable and Helmholtz coil systems inside. The lab reference frame in spherical coordinates is sketched in white here and in Figure 2a.
(c) Four cesium vapor cells on a common glass substrate [24]. This assembly is sawed along the dashed lines to get the two separated vapor cells. Both are equipped with a ceramic thin-film heater on one silicon side wall. One other silicon side wall of one cell is blackened with soot, where the fiberized temperature sensor is pressed against.
(d) Schematic drawing of the complete measurement setup. PL: pumping laser; AM: amplitude modulator; CL: collimating lens; LP and λ/4: combination of linear polarizer and quarter wave plate; NBS: non-polarizing 50:50 beam splitter; VC: Cs vapor cells; PD: photodiodes; I/U: transimpedance preamplifiers; SA: summing amplifier; LI: lock-in amplifier with integrated generator; TA: tuneable amplifier.

### 3.2. Measurement Procedure

The measurements covered the complete upper hemisphere of the lab coordinate frame (see Figure 2a,b). Firstly, a polar angle θ from the set [0; 15; 30; 45; 60; 75; 90°] was fixed. Then, the azimuth angle φ was varied manually between −165° and 165° in steps of 15° (due to the connection of the pull strings, the value of angle φ = 180° is not accessible). To exclude the influences of temporal parameter drifts, φ was increased in steps of 30° from 0° up to 150°, decreased from 165° to −165°, and finally once again increased from −150° to 0°, all repeated in steps of 30°. Then, the polar angle was tuned to the next value by adjusting the magnetic field direction in the *xz* plane by applying different currents to *x* and *y* coils. After each such change, the shielding barrel was demagnetized to minimize residual magnetic fields in the μ-metal shielding before the next series with varying azimuth φ started. For the demagnetization, a 10 Hz magnetic field with slowly decreasing amplitude (ramping fully down within 1 min) was generated inside the shieling assembly. At each position given by [φ,θ], three resonance curves around the Larmor frequency were independently recorded for the individual magnetometers #1 and #2 as well as for their sum (in the following labeled as #12).

The measurements were carried out at fixed lock-in phase offset, determined once in the (best) sensor position when ***k*** was perpendicular to ***B***_0_. This method resembles the operational mode in a real-world geophysical measurement application when the OPM sensor is moved and rotated within the background magnetic field. The insensitivity of the lock-in phase on the sensor orientation was checked carefully.

All relevant parameters for the valuation of the OPM operation were taken from the dispersive (*Y*) lock-in signal. One example is shown in Figure 3. The linear fit around resonance delivers the conversion factor d*Y*/d*f*, which is referred to as signal size *S*. This is done because in our IM operation of the OPM, the absorptive (*X*) lock-in signal shows a large amplitude offset due to the simultaneous use of the modulated laser beam for both pump and probe [21]. The zero crossing of the measured *Y* signal delivers the Larmor frequency *f*_L_ = γ·*B*_0_ as the measure of the magnetic field *B*_0_ (with γ = 3.5 Hz/nT being the gyromagnetic ratio of cesium). The signal shown in Figure 3 is not completely symmetric because the pure pumping light modulation (without magnetic-field resonance) already has a minute inherent frequency dependence. This, however, is identical for all measurements. For that reason, a lock-in phase was so chosen (and kept constant for all measurements) that the wings far away from the resonance are symmetrical around zero. We verified that any potentially slight maladjustment in the lock-in phase had only a minor impact on the determined Larmor frequency as long as the signals were not too small. Hence, we have omitted the data for the incident angles near 0° and 180° in the later discussion about measured Larmor frequencies.

The data variation given in the example in Figure 3 has a typical size valid for all measurements. Resulting error bars are only shown in the first Figure of Section 4. In all subsequent diagrams, they are omitted for the sake of clarity. In all these cases, the variances are comparable to the size of the data symbols.

**Figure 4:**
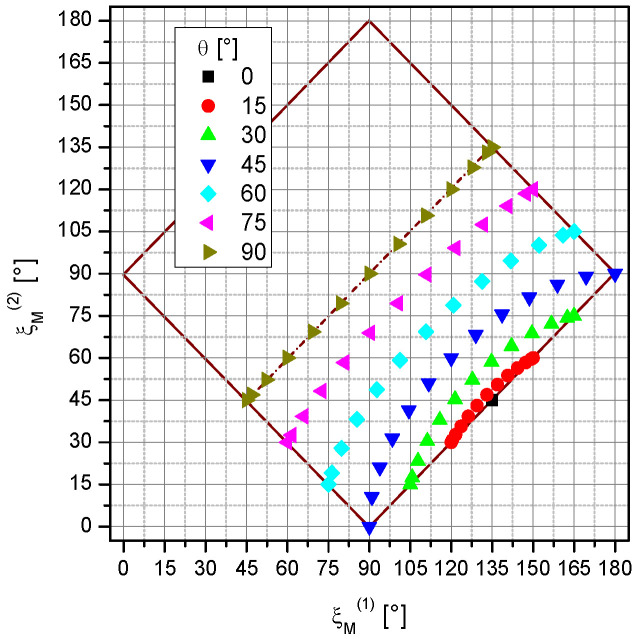
Incident angles ξ_M_^(2)^ vs. ξ_M_^(1)^ of channels #2 and #1 met in the measurement situations. The different colors represent the polar angle θ. The individual data points within these series cover all the measured azimuth values φ. Pairs of incident angles exist only in the framed range. The part above the symmetry line, belonging to polar angles between 0° and −90°, was not covered because it is symmetrical to the measured data.

These data were taken in the lab system with the azimuth angle φ and the polar angle θ. For easier interpretation, we transformed the data into the coordinates of the magnetometer system φ_M_ and θ_M_. This transformation, as described in Appendix A, is necessary due to the ±45° inclined installation of the cells (Figure 2a). In the Appendix A, the derivation of the incident angle ξ_M_, the angle between pumping light direction ***k*** and magnetic field ***B***_0,_ is also given. It is a convenient one-dimensional parameter that we use to interpret the measurement results. The detailed values of the incident angles of the two channels accessible with our measurement configuration in the lab system are shown in Figure 4.

## 4. Results and Discussion

**Figure 5:**
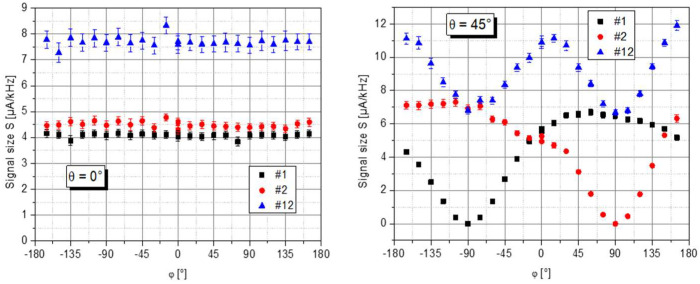
Measured signal size *S* of the individual magnetometers #1 and #2 and their sum #12 in dependence on the lab frame’s azimuthal angle φ. (**Left**) and (**Right**): for a lab-frame polar angle θ of 0° and 45°, respectively.

Figure 5 shows data in the lab system recorded for a full rotation of the turntable at θ = 0° and θ = 45°. As expected from Equation (4), at θ = 0°, the signal size *S* is largely independent of the azimuth angle φ. In contrast, at θ = 45° channels #1 and #2 cover the full range from 0° to 180° of the incident angle ξ between ***k*** and ***B***_0_. Thus, *S* changes strongly, but in alternating fashion. It should be mentioned that the signal of channel #1 is about 10% smaller than that of signal #2. Such a difference can be expected since creating cesium and nitrogen buffer gas is a unique process in the cells (cf. their different nitrogen pressure reported in Section 3.1). However, this does not degrade the functionality of the two-cell setup, as the sum signals #12 in Figure 5 show.

### 4.1. Signal Size and Magnetic Field Resolution

**Figure 6:**
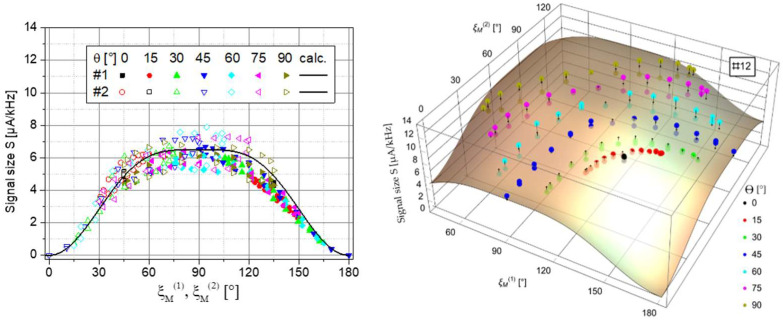
Signal sizes. (**Left**): Measured data of the single channels #1 and #2 vs. their respective incident angle. The calculated curve displays Equation (5). (**Right**): Measured data of the sum channel #12 in dependence on the two respective incident angles.

In the magnetometer system, the signal size *S* has a dependence on the incident angle ξ_M_, given by:

(5) $$S(\xi_{M})=1-{cos}^{4}(\xi_{M})$$

which generalizes Equation (4) with the angle ξ_M_ between light propagation direction ***k*** and the direction of the magnetic field ***B***_0._ The left side of Figure 6 shows that, in fact, both individual channels follow this expected dependence, whereas, in the summing channel #12, the polar dead zones of the individual channels are canceled as desired (right side of Figure 6).

**Figure 7:**
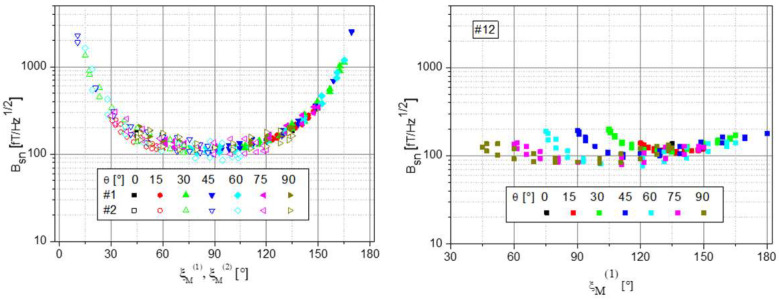
Shot-noise limited magnetic-field resolution. (**Left**): Measured data of the individual channels #1 and #2 vs. their respective incident angle. (**Right**): Measured data of the sum channel #12. For good visibility, the data are shown solely depending on incident angle #1.

This signal behavior of the single channels #1 and #2 and the sum channel #12 is also reflected in the shot-noise limited magnetic-field resolution:

(6) $$B_{\mathrm{sn}}=\frac{I_{\mathrm{sn}}}{\gamma \cdot \left|dY/df\right|},$$

with $I_{sn}=\sqrt{2\cdot e\cdot I_{dc}}$ being the shot noise of the DC photocurrent *I*_dc_ (in our case, the mean value of the square-wave modulated pumping light). As Figure 7 shows, the loss in sensitivity near the dead zones of the single channels is largely canceled in the summing channel. In the worst case, the total sensor sensitivity drops by a factor of two. In the best case (ξ_M_ at or near 90° simultaneously for both channels), it improves by factor 1/√2. This is because even though the noise *I*_sn_ increases by factor 1/√2, the slope d*Y*/d*f* doubles.

Therefore, the sensor setup with two orthogonally-pumped intensity-modulated OPM cells enables dead-zone-free sensor operation.

### 4.2. Sensor Field Readings

**Figure 8:**
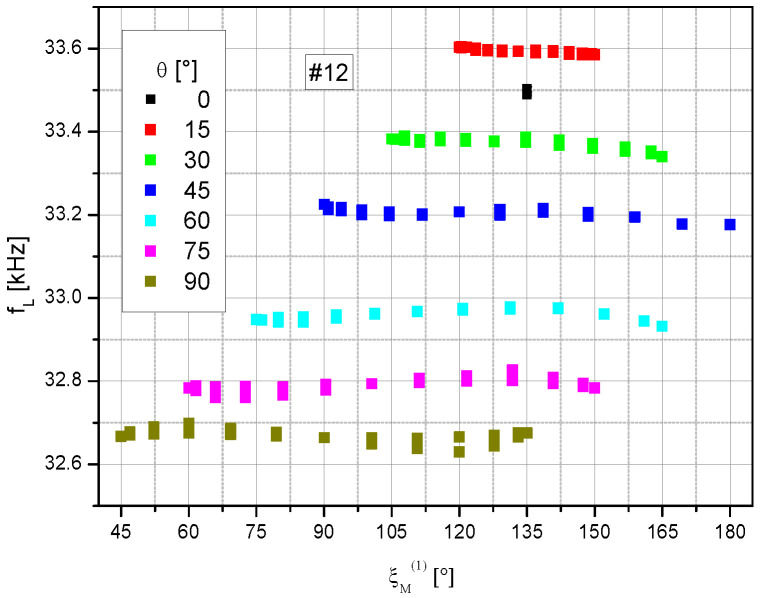
Raw Larmor frequency readings of the sum channel #12 for different polar angles θ.

The raw sensor magnetic field readings, given by the measured Larmor frequencies (cf. Section 3.2), show large offsets between the series with fixed polar angle θ (Figure 8). These offsets may be either due to residual fields in the shielding, introduced by the change of the ***B***_0_ field direction (despite the procedure described in Section 3.2), or slight differences in the coil calibration constants of the *x* and *z* magnetic field coil pairs (cf. Figure 2b). Subsequently, the offsets of the Larmor frequencies between the series were eliminated. For this purpose, all individual measurement series were shifted in such a way that data around ξ_M_^(1)^ = 135°, which are part of all measurement series (cp. Figure 4), are transferred to the θ = 45° series. To maintain their mutual reference, this shift was applied uniformly to all data from the three channels #1, #2, and #12.

**Figure 9:**
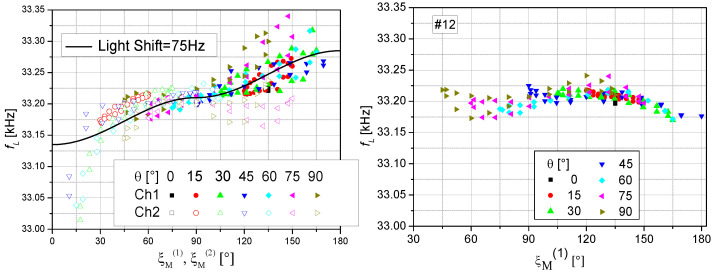
Corrected Larmor frequency readings. (**Left**): Measured data of channels #1 and #2 vs. their respective incident angle, together with a modeled dependence due to a vector light shift with an effective magnetic field strength corresponding to 75 Hz and pointing along ***k*** directions of each channel, respectively. This light shift value was calculated with the formalism presented in [3], using the detuning of the laser frequency and the pump power applied in Section 3.1. (**Right**): Measured data of the sum channel #12 vs. incident angle #1.

These corrected data are shown in Figure 9. In the ideal case, one would expect the same Larmor frequency independent of the measurement situation. However, individual channels 1 and 2 especially show various systematic deviations. The increasing falsification for incident angles ξ_M_ < 30° and >150° is due to the respectively small or zero signal size in these dead zones (cf. Figure 6).

The inspection of the remaining dependence of the data hints at a systematic shift introduced by the vector light shift (LS) effect. The vector light shift can emerge when circularly polarized light is detuned from the involved optical transitions. In our parameter set, the laser frequency was slightly detuned for the best magnetic-field resolution (see Section 3.1). The light shift effect can be understood as a virtual magnetic field pointing along the photon spin vector of the light, which adds vectorially to the external magnetic field ***B***_0_. In this way, the LS can lead to shifts depending on sensor orientation. For the two opposite helicities of the circularly polarized light, the magnetic field sensor readings result from:

(7) $$B_{\sigma +}^{2}=B_{0}^{2}+2\cdot B_{0}\cdot LS(\xi )\cdot \cos\xi +LS(\xi {)}^{2}\;B_{\sigma -}^{2}=B_{0}^{2}-2\cdot B_{0}\cdot LS(\xi )\cdot \cos\xi +LS(\xi {)}^{2},$$

where *LS*(ξ) follows the dependence:

(8) $$LS(\xi )=\mathrm{LS}\cdot \left|\cos\xi \right|,$$

because the light shift is not only proportional to the pumping rate but also to the degree of circular polarization [25]. The light shift effect vanishes completely at ξ = 90°, where the intensity-modulated OPM also has optimal sensitivity. This is an additional advantage of this operational mode. The addition of the signals of the two orthogonally arranged cells eliminates the pitfall of dead zones and also reduces the systematic shift of Larmor frequency due to LS partially, but not completely (Figure 9 right).

A straightforward and common solution to the LS problem is to use the average of the signals from two cell compartments with identical parameters, interrogated with circularly polarized light of opposite helicity [3]. With this arrangement, the nonlinear Zeeman effect, which becomes significant when working in magnetic fields *B*_0_ of geomagnetic strength (viz. around 50 µT in central Europe), can also be suppressed. This demands as good as possible tuned pump power of the two beams with opposite helicity.

## 5. Summary and Conclusions

With an assembly of two vapor cells pumped by the same intensity-modulated light but perpendicularly propagating laser beams, a (dead-zone free) omnidirectional OPM can be implemented. This concept especially leverages the strengths of the Bell-Bloom magnetometer: It has a beneficially wide range of sensor orientations offering high sensitivity perpendicular to the pump direction. The signals can be directly added after passing through the cells. Thus, only a single-channel signal processing is needed. When light propagation direction and magnetic field vector are oriented perpendicular, the intensity-modulated OPM shows the best sensitivity while, at the same time, the vector light shift is absent. However, in other directions, the vector light shift and the nonlinear Zeeman effect cause systematic shifts in the sensor’s magnetic field readings. These detrimental effects could be compensated to a high degree when using two vapor cell compartments in each of the two laser propagation directions, interrogated by two beams with circularly polarized light of opposite helicity. However, a balancing of all the operational parameters to a very high degree is indispensable. The experimental proof of this concept is a future research topic.

## Data Availability

The data generated and analysed during the current study are available from the corresponding author on reasonable request.

## Appendix A

The transformation of the data from the lab system with azimuth angle φ and polar angle θ to the magnetometer system φ_M_ and θ_M_ is performed as follows:

The Cartesian coordinates *x*, *y*, *z* in the lab system are connected to the spherical basis by:

(A1) x(φ,θ)=sin⁡(θ)⋅cos⁡(φ)y(φ,θ)=sin⁡(θ)⋅sin⁡(φ)z(φ,θ)=cos⁡(θ).

Due to the ±45° inclined installation of the cells on the rotation axis of the turntable (Figure 2a), consecutive mathematical rotations of β = 90° about the *x*-axis and α = −45° about the *y*-axis are needed. Thus, the coordinates *x*_M_, *y*_M_, and *z*_M_ in the magnetometer system calculate from their counterparts in the lab system with the transformation:

$$\left(\begin{array}{c} x_{M}(\varphi ,\theta )\\ y_{M}(\varphi ,\theta \\ z_{M}(\varphi ,\theta ) \end{array}\right)=\left(\begin{array}{ccc} cos(\beta ) & 0 & sin(\beta )\\ 0 & 1 & 0\\ -sin(\beta ) & 0 & cos(\beta ) \end{array}\right)\cdot \left(\begin{array}{ccc} 1 & 0 & 0\\ 0 & cos(\alpha ) & -sin(\alpha )\\ 0 & sin(\alpha & cos(\alpha ) \end{array}\right)\cdot \left(\begin{array}{c} x(\varphi ,\theta )\\ y(\varphi ,\theta )\\ z(\varphi ,\theta ) \end{array}\right)$$

With the fixed rotation angles given above, this equation is modified as:

(A2) $$x_{M}(\varphi ,\theta )=\frac{1}{2}\sqrt{2}\cdot \left[y(\varphi ,\theta )+z(\varphi ,\theta )\right]\;y_{M}(\varphi ,\theta )=\frac{1}{2}\sqrt{2}\cdot \left[y\left(\varphi ,\theta \right)-z(\varphi ,\theta )\right]\;z_{M}(\varphi ,\theta )=-x(\varphi ,\theta ).$$

Finally, the angles in the magnetometer system are given by:

(A3) $$\varphi_{M}(\varphi ,\theta )=\mathrm{atan}2\left[x_{M}(\varphi ,\theta ),y_{M}(\varphi ,\theta )\right]\;\theta_{M}\left(\varphi ,\theta \right)=\mathrm{acos}\left[z_{M}\left(\varphi ,\theta \right)\right].$$

Therein, the function atan2 eliminates the ambiguity of the common atan function.

The incident angle ξ, the angle between pumping light direction ***k*** and magnetic field ***B***_0,_ can be inferred from the great-circle (or orthodromic) distance, where the arc length of a distance on a great circle is described by the angle between its two endpoints [26]. Translated to our configuration, this incident angle is calculated as:

(A4) $$\xi_{M}(\varphi_{M},\theta_{M})=\mathrm{acos}\left[\sin(\theta_{M})\cdot \cos(\varphi_{M})\right].$$

Since this expression is ambiguous due to the cos function, we incorporated the additional convention that ξ_M_ is between 0 and 90° when the pumping direction ***k*** has a component parallel to ***B***_0_ and that it is between 90° and 180° for an antiparallel component.
